# Hospital Construction

**Published:** 1897-12-04

**Authors:** 


### HOSPITAL CONSTRUCTION.

### THE PRINCE ALFRED HOSPITAL, SYDNEY.

The managers of the Prince Alfred Hospital at Sydney have recently reorganised their operating theatre, and among the improvements introduced are some which are worthy of notice.

The floor is of strong Belgian tiles, laid with a slight incline towards one side of the arena, where it ends in an open gutter two inches from the wall. This gutter is cut in long lengths of thick slate, with a good fall towards each end, where it terminates in well ventilated traps. The floor is laid continuously without any break or opening, all pipes being attached to the walls and entering above the floor line, so that for cleansing purposes there is an unbroken surface. Our only criticism in regard to this floor is that where really hard cement is available, it is a great advantage to have a continuous flooring rather than one formed of separate tiles, but there is no doubt that the method of laying out the floor above described is the correct one. We should, however, have preferred to have only one trap. The pipes are of copper nickel-plated, the object of this being that they may be cleaned without the use of plate-powder, and thus without the production of dust. The earthenware sinks are placed over the above-mentioned gutter, into which all the wastes discharge. Each basin is supplied with hot and cold sterilised water, and all the taps are worked by the foot, so that the hand shall not be infected by contact with a dirty tap. The arrangement for the supply of sterilised water is peculiar, and, if it should turn out to be efficient, is one of considerable interest. Of course, it is easy enough to get hot sterilised water; the difficulty is with the cold supply. This has been got over in the following manner: Careful biological investigation having shown that the hot water supply of the hospital was sterile, a copper tank containing 400 gallons was built and placed in the room over the anæsthetic-room, and from this pipes have been led to supply cool water to the range of basins in the theatre. The fittings of this tank have been so arranged that it feeds itself with hot, and therefore sterile, water at the top when cool water is drawn off from the bottom; thus it is always full, and the hot water by which it is fed has time to cool before it is wanted. The tank is also furnished with a steam-coil by which it can at any time be brought to the boiling point when the resterilisation of the tank and its contents is found necessary. The steriliser for the instruments is placed in the theatre, but a larger one for dressings, towels, surgeon's white coats and aprons, &c., is placed in an adjoining room. Both are heated by steam. Among other arrangements for avoiding dust, we would draw attention to the fact that all the blinds are placed outside the windows, although worked by cords from within, a principle which might be much more largely adopted than it is.

